# Magnesium Ions Inhibit the Expression of Tumor Necrosis Factor α and the Activity of γ-Secretase in a β-Amyloid Protein-Dependent Mechanism in APP/PS1 Transgenic Mice

**DOI:** 10.3389/fnmol.2018.00172

**Published:** 2018-05-30

**Authors:** Xin Yu, Pei-Pei Guan, Di Zhu, Yun-Yue Liang, Tao Wang, Zhan-You Wang, Pu Wang

**Affiliations:** College of Life and Health Sciences, Northeastern University, Shenyang, China

**Keywords:** magnesium ions, tumor necrosis factor α, presenilin enhancer 2, nicastrin, β-amyloid protein

## Abstract

Alzheimer’s disease (AD) is a neurodegenerative disease characterized by cognitive impairment. The neuropathological features of AD are the aggregation of extracellular amyloid β-protein (Aβ) and tau phosphorylation. Recently, AD was found to be associated with magnesium ion (Mg^2+^) deficit and tumor necrosis factor-alpha (TNF-α) elevation in the serum or brains of AD patients. To study the relationship between Mg^2+^ and TNF-α, we used human- or mouse-derived glial and neuronal cell lines or APP/PS1 transgenic (Tg) mice as *in vitro* and *in vivo* experimental models, respectively. Our data demonstrates that magnesium-L-threonate (MgT) can decrease the expression of TNF-α by restoring the levels of Mg^2+^ in glial cells. In addition, PI3-K/AKT and NF-κB signals play critical roles in mediating the effects of Mg^2+^ on suppressing the expression of TNF-α. In neurons, Mg^2+^ elevation showed similar suppressive effects on the expression of presenilin enhancer 2 (PEN2) and nicastrin (NCT) through a PI3-K/AKT and NF-κB-dependent mechanism. As the major components of γ-secretase, overexpression of presenilin 1 (PS1), PEN2 and NCT potentially promote the synthesis of Aβ, which in turn activates TNF-α in glial cells. Reciprocally, TNF-α stimulates the expression of PEN2 and NCT in neurons. The crosstalk between TNF-α and Aβ in glial cells and neurons could ultimately aggravate the development and progression of AD.

## Introduction

Magnesium ions (Mg^2+^) were recently found to be downregulated in the brain tissue of Alzheimer’s disease (AD) patients compared to those of healthy controls, especially in the hippocampus (Cilliler et al., 2007). Interestingly, preclinical studies have shown that MMFS-01, a derivative compound of magnesium-L-threonate (MgT) was effective in alleviating cognitive decline in aging rodents (Liu G. et al., 2015). As a synapse density enhancer (Slutsky et al., 2004; Zhou and Liu, 2015), the elevation of brain Mg^2+^ prevented synaptic loss and reversed cognitive deficits in AD mouse model (Li et al., 2014). Indeed, Mg^2+^ restoration attenuated memory impairment by activating protein kinase C (PKC) in experimental animals (Olariu et al., 2002; Libien et al., 2005). More notably, our prior work revealed that Mg^2+^ treatment enhanced clearance of Aβ in an APH-1α/1β-dependent manner in APP/PS1 transgenic (Tg) mice (Yu et al., 2015). All of this evidence points to the fact that Mg^2+^ influx is beneficial for treating AD.

Following these observations, particular attention has been given to the blockage effects of Mg^2+^ on the composition and function of NMDA receptors (Slutsky et al., 2004), whose expression is critical for neuroinflammation during the course of AD development and progression (Lee et al., 2011). As NMDARs are able to regulate the learning and long-term memory of *Drosophila* (Miyashita et al., 2012), it is easy to speculate that Mg^2+^ is potentially responsible for altering the memory in AD by affecting neuroinflammation. Supporting this hypothesis, Mg^2+^ elevation was reported to inhibit the expression of inflammatory markers, such as tumor necrosis factor-α (TNF-α) in hypothyroid rats (Abbas and Sakr, 2016). Importantly, MgT treatment clearly restored the short-term memory deficits induced by spared nerve injury in aging animals (Wang et al., 2013). Although these observations did not extend to Aβ production, a number of studies have shown that TNF-α was responsible for impairing memory by accelerating the abnormal cleavage of APP during the course of AD development and progression. For example, Alvarez et al. (2007) reported that the levels of TNF-α were elevated in AD patients. Interfering with the metabolism of TNF-α results in a decrease in behavioral impairments in an AD mice model (Giuliani et al., 2009). Importantly, anti-TNF-α reduces the production of Aβ and phosphorylation of tau in APP/PS1 Tg mice (Shi et al., 2011). These observations have been further supported by randomized clinical trials (Tobinick, 2009).

Although Mg^2+^ likely regulates the production of Aβ by inhibiting the expression of TNF-α, the underlying mechanisms remain unknown. To understand the functional significance of Mg^2+^ in the production of Aβ, we determined its roles in the expression of γ-secretase. Using MgT as a model drug, we found that MgT decreases the expression of TNF-α in glial cells. In neurons, MgT showed suppressive effects on the expression of presenilin enhancer 2 (PEN2) and nicastrin (NCT). By decreasing the crosstalk between TNF-α and PEN2/NCT, MgT achieved therapeutic effects against AD.

## Materials and Methods

### Reagents

Aβ_1–42_, LY294002 and Quinazoline (QNZ) were obtained from Sigma-Aldrich Corp (St. Louis, MO, USA); MgT was purchased from Soyoung Biotechnology Company (Shanghai, China). Antibodies specific for β-actin, AKT, p-AKT, NF-κB, p-NF-κB, NCT, PEN2, TNF-α, NeuN, GFAP, Alexa Fluor 488-labeled, Alexa Fluor 555-labeled, and HRP-labeled secondary antibody were purchased from Cell Signaling Technology, Inc., (Danvers, MA, USA). Iba-1 antibody was obtained from Merck Millipore (Bedford, MA, USA). DAPI was obtained from Beyotime Institute of Biotechnology (Haimen, JS, China). All reagents for the qRT-PCR and SDS-PAGE experiments were purchased from Bio-Rad Laboratories. All other reagents were from Invitrogen (Carlsbad, CA, USA) unless otherwise specified.

### Animal Committee

All animals were handled according to the care and use of medical laboratory animals (Ministry of Health, Peoples Republic of China, 1998) and all experimental protocols were approved by the Laboratory Ethics Committees of China Medical University and College of Life and Health Sciences of Northeastern University.

### Transgenic Mice and Treatments

APP/PS1 transgenic mice [B6C3-Tg (APPswe, PSEN1dE9) 85Dbo/J (Stock Number: 004462)] (Tg) and wild-type C57BL/6 mice (WT) were originally obtained from The Jackson Laboratory (Bar Harbor, ME, USA). Genotyping was performed 3–4 weeks after birth. The mice were kept in cages in a controlled environment (22–25°C, relative humidity, 12-h light/dark cycle with free access to food and water). In select experiments, mice at the age of 4 months were treated with Mg^2+^ (100 mg/kg/d) in drinking water (4 mg/ml) for 2 months before the expression of TNF-α, PEN2 and NCT was determined. For determining the production of Aβ, GFAP and Iba-1, mice at the age of 4 months were treated with Mg^2+^ (100 mg/kg/d) in drinking water (4 mg/ml) for 5 months. The average drinking water consumed per day was monitored (~6.4 ml/d) and the body weight of the mice was ~20 g. All efforts were made to minimize animal suffering and the number of animals used. The brains of mice in different groups were collected under anesthesia and perfusion-fixed as previously described (Yu et al., 2015; Wang et al., 2016a,b, 2017).

### Aβ_1–42_ Oligomer Preparation

The method for preparing oligomers Aβ_1–42_ (oAβ) has been described previously (Yu et al., 2015; Wang et al., 2017). In brief, Aβ_1–42_ protein (Stock Number: A9810, Sigma, St. Louis, MO, USA) was freeze-drying in monomer form after dilution to a final concentration of 1 μg/μl in 100% hexafluoroisopropanol (HFIP) and the solution was aliquoted into sterile Eppendorf tubes. HFIP was then evaporated under vacuum and the peptide was stored at −20°C before reconstitution. For preparing oAβ, the peptide was initially resuspended in dimethyl sulfoxide (DMSO) at 20 μg/μl with water bath ultrasonication for 10 min and the solution was then diluted to a final concentration of 0.4 μg/μl in phenol red-free F-12 media and incubated at 4°C for 24 h.

### Intracerebroventricular Injection

MgT, TNF-α, oAβ or vehicle were injected (i.c.v.) into WT mice as previously described (Yu et al., 2015; Wang et al., 2016a,b, 2017). Briefly, stereotaxic injections were placed at the following coordinates from bregma: mediolateral: −1.0 mm; anteroposterior: −0.22 mm; dorsoventral: −2.8 mm. Following injection, each mouse recovered spontaneously on a heated pad. The reliability of injection sites was validated by injecting trypan blue dye. Twenty-four hours post-injection, the mice were anesthetized and perfused, and the brains were collected.

### Cell Culture

Human glioblastoma A172, neuroblastoma SH-SY5Y and mouse astrocytes D1A, microglia BV2 and neuroblastoma n2a cells were grown (37°C and 5% CO_2_) on 6 cm tissue culture dishes (10^6^ cells per dish) in the appropriate medium. In a select set of experiments, the cells were grown in serum-free medium for an additional 24 h before incubation with inhibitors in the absence or presence of MgT, as previously described (Wang et al., 2014b). In separate experiments, the primary cultured astrocytes were separated from early postnatal (P0-P1) mouse hippocampus and cortex as previously described (Beaudoin et al., 2012; Xu et al., 2014). In separate experiments, astrocytes and neurons were cocultured using transwells, in which astrocytes were seeded in the upper chamber and neurons in the lower chamber of the transwell. After 24 h, the neurons were immunostained with a TNF-α antibody as previously described (Wang et al., 2017).

### Quantitative Real-Time PCR (qRT-PCR)

QRT-PCR assays were performed with the MiniOpticon Real-Time PCR detection system (Bio-Rad) using total RNA and the GoTaq one-step real-time PCR kit with SYBR green (Promega) and the appropriate primers as previously described (Wang et al., 2010a,b, 2011a,b, 2012). The GenBank accession number and forward and reverse primers for human and mouse GAPDH were provided in our previous publications (Wang et al., 2014a; Guan et al., 2015a,b); for human TNF-α F-TCCTTCAGACACCCTCAACC, R-AGGCCCCAGTTTGAATTCTT; for mouse TNF-α F-AGCCCCCAGTCTGTATCCTT, R-ACAGTCCAGGTCACTGTCCC, for human PEN2 F-GCTATGAACCTGGAGCGAGTG, R-GAAGGAGAGGTAGTCCCCAAGG; for mouse PEN2 F-CGTGATCTTGCGTCTGTCAT, R-AACGCCTCTCTGAAGAACCA; for human NCT F-TAGCCTAGAGAGGCCGCTAACAGA, R-GCTTGCTCTCCAGCAGAACCATGT; for mouse NCT F-GCTTCAGCACCCTTGTCTTC, R-TAAGCAGGCCCAGAGACAGT. The gene expression values were normalized to those of GAPDH.

### Western Blot Analysis

Tissues or cells were lysed in radio-immune precipitation assay buffer (25 mM Tris-HCl [pH 7.6], 150 mM NaCl, 1% NP-40, 1% sodium deoxycholate and 0.1% SDS) containing protease inhibitor cocktail (Pierce Chemical Company). The protein content of the cell lysates was determined using a bicinchoninic acid (BCA) protein assay reagent (Pierce Chemical Company). The total cell lysates (4 μg) were subjected to SDS-PAGE, transferred to a membrane, and probed with a panel of specific antibodies. Each membrane was only probed with one antibody. β-actin was used as a loading control. All western hybridizations were performed at least in triplicate using a different cell preparation each time.

### Immunohistochemistry

Mouse brains were collected from WT or APP/PS1 Tg mice and immobilized with 4% paraformaldehyde. Serial sections (10 μM thick) were cut using a cryostat (Leica, CM1850, Germany). Slides were first rehydrated in a graded ethanol series and submerged in 3% hydrogen peroxide to eliminate endogenous peroxidase activity. TNF-α levels were determined using an immunohistochemical staining kit, following the manufacturer’s instructions (Invitrogen, Carlsbad, CA, USA). In selected experiments, the slices from the brains of human and mouse were double-stained with TNF-α (Alexa Fluor 488-labeled secondary IgG) and NeuN (Alexa Fluor 555-labeled secondary IgG) antibody as previously described (Wang et al., 2017).

### Human Brain Samples

Human brain samples were obtained with informed consent from the families and with the ethical approval of institutional Ethics Committees from New York Brain Bank at Columbia University (Alzheimer Disease Research Centre, Taub Institute), New York, USA, and Lund University (Elisabet Englund, Dnr 286–2014), Lund, Sweden. Experiments were performed in accordance with the guidelines approved by Department of Experimental Medical Science at Lund University, Lund, Sweden and Department of Biology and Biological Engineering at Chalmers University of Technology, Göteborg, Sweden. All subjects gave written informed consent in accordance with the Declaration of Helsinki. All samples were taken from the prefrontal cortex, more specifically from gyrus rectus. The brain samples were fixed with formaldehyde solution, sectioned into 40 μm thick slices and kept in phosphate buffered saline (PBS). The serial numbers were TT4263 (early stage of AD: the patient was a 73-year-old man who was diagnosed as a mild AD patient), T4308 (middle stage of AD: the patient was an 86-year-old man who was diagnosed as a moderate AD patient), T4339 and T4304 (late stage of AD: the patients was an 88-year-old woman and an 84-year-old woman who were diagnosed as severe and end-stage AD patients).

### Statistical Analysis

All data are represented as the mean ± SE of at least three independent experiments. The statistical significance of the differences between the mean values was determined with the Student’s *t*-test, 1-way or 2-way analysis of variance (ANOVA), as appropriate (Wang et al., 2016a).

## Results

### TNF-α, PEN2 and NCT Are Markedly Upregulated During the Course of AD Development and Progression

**Figure 1:**
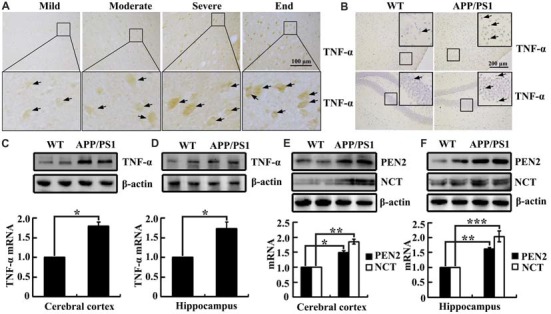
Tumor necrosisfactor-α (TNF-α), presenilin enhancer 2 (PEN2) and nicastrin (NCT) are markedly upregulated in Alzheimer’s disease (AD) patients and APP/PS1 Tg mice. **(A)** The tissue blocks of human brains at different stages of AD were collected from the New York Brain Bank at Columbia University. Forty micrometer free-floating slices were prepared using a cryostat (*n* = 1). **(B–F)** The brains of 3-month-old APP/PS1 transgenic mice were collected after anesthesia and perfusion (*n* = 8). **(A,B)** The immunoreactivity of TNF-α was determined by immunohistochemistry using an anti-TNF-α antibody. These images are representative of eight independent experiments and one sample patient, all with similar results. **(C–F)** mRNA and protein levels of TNF-α, PEN2 and NCT were determined by qRT-PCR and western blot analysis, GAPDH and β-actin served as the internal control. The data represent means ± SE of three independent experiments. *p < 0.05, **p < 0.01 and ***p < 0.001 compared with the WT mice.

As shown in (Figure 1A), TNF-α immunostaining was evident in the human cerebral cortex, and the positive staining gradually increased with the progression of AD. In accordance with these data, TNF-α immunostaining was also induced in the cerebral cortex and the dentate gyrus (DG) region of the hippocampus in 3-month-old APP/PS1 transgenic mice compared with WT C57BL/6 mice (Figure 1B). To further verify this hypothesis, we examined the mRNA and protein levels of TNF-α in the above AD model. In agreement with the immunostaining data, our experiments revealed that mRNA and protein levels of TNF-α were upregulated in the cerebral cortex and the hippocampus of the APP/PS1 Tg mice compared with that of the WT mice (Figures 1C,D). In addition, we found that the mRNA and protein levels of PEN2 and NCT were also upregulated in the cerebral cortex and the hippocampus of the above AD model (Figures 1E,F). Collectively, these observations reinforce the possible involvements of TNF-α, PEN2 and NCT in aggravating AD.

**Figure 2:**
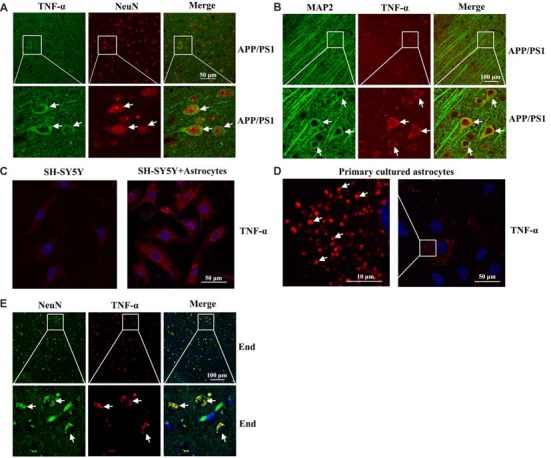
Localization of TNF-α in the brains of AD patients and APP/PS1 Tg mice. **(A,B)** The brains of 3-month-old APP/PS1 Tg mice were collected after anesthesia and perfusion (*n* = 10). The slices of mouse brains were double-stained with NeuN (red) and TNF-α (green) **(A)** or MAP2 (green) and TNF-α (red) antibody **(B)** before observation using confocal microscopy. **(C)** The astrocytes were co-cultured with neurons using transwell experiments for 24 h. The neurons were then immunostained with TNF-α antibody before observation under confocal microscopy. **(D)** Primary cultured astrocytes were immunostained with a TNF-α antibody before observation under confocal microscopy. **(E)** The tissue blocks of human brains at a late stage of AD were collected from the New York Brain Bank at Columbia University. Forty micrometer free-floating slices were prepared using a cryostat (*n* = 1). The slices of human brains were double-stained with NeuN (green) or TNF-α (red) antibody before observation using confocal microscopy.

To further determine the localization of TNF-α immunostaining experiments were carried out. As a consequence, we found that TNF-α staining was predominantly colocalized with NeuN or MAP2 in the brain of 3-month-old APP/PS1 Tg mice (Figures 2A,B). However, our results further demonstrated that the expression of TNF-α was relative low in neurons (Figure 2C). By these observations, we could still not exclude the possibility that TNF-α is secreted from astrocytes, which migrate and bind to their receptors on neurons as a type of secreted cytokine. To further verify this hypothesis, we used transwell experiments to co-culture astrocytes and neurons. The antibody-specific for TNF-α was used to determine the level of TNF-α in neurons. Interestingly, the level of TNF-α was elevated in neurons by co-culturing with astrocytes, which indicated the enrichment of TNF-α in astrocytes (Figure 2C). As expected, we found that TNF-α was highly expressed in primary cultured astrocytes (Figure 2D). To further validate the above observations, TNF-α also colocalized with NeuN in the brains of AD patients (Figure 2E). Therefore, we demonstrated that TNF-α might translocate from the astrocytes to the neurons, which shows its existence in neurons.

### Mg^2+^ Influx Attenuates the Synthesis of TNF-α, PEN2 and NCT in Glial and Neurons

**Figure 3:**
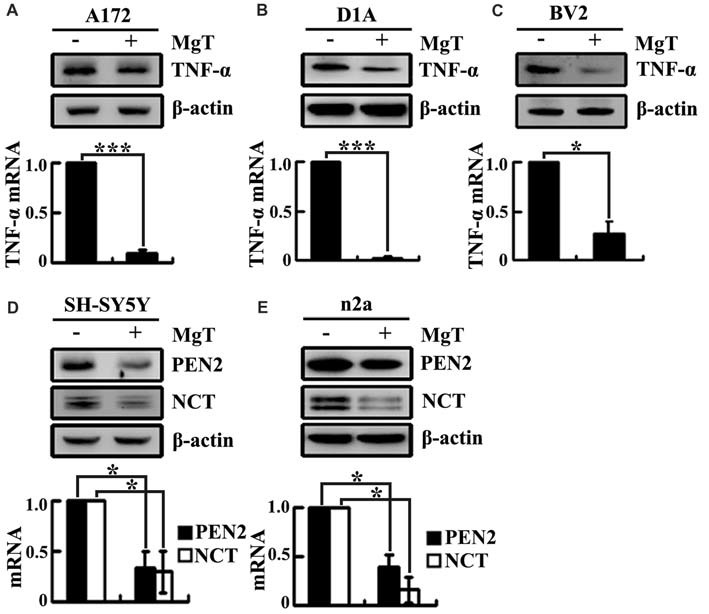
Mg^2+^ elevation suppresses the expression of TNF-α, PEN2 and NCT in cultured neurons and glial cells. Human glioblastoma A172 **(A)**, mouse astrocytes D1A **(B)** or microglia BV2 cells **(C)** were treated with MgT (50 μM) for 48 h; TNF-α mRNA and protein levels were determined by qRT-PCR and western blots. GAPDH and β-actin served as the internal control. In select experiments, human or mouse neuroblastoma SH-SY5Y **(D)** and n2a **(E)** cells were treated with MgT (50 μM) for 48 h. mRNA and protein levels of PEN2 and NCT were determined by qRT-PCR and western blots. GAPDH and β-actin served as the internal control. The data represent means ± SE of three independent experiments. *p < 0.05 and ***p < 0.001 compared with the vehicle-treated controls.

Our prior work demonstrated that the levels of Mg^2+^ were reduced in the CSF of the APP/PS1 Tg mice (Yu et al., 2015). These observations implied the possible protective effects of Mg^2+^ against AD. To further elucidate the ability of Mg^2+^ to regulate the expressions of TNF-α, PEN2 and NCT in glia or neurons, we treated human A172 cells with MgT (50 μM). As shown in Figure 3A, MgT treatment markedly suppressed the mRNA and protein levels of TNF-α in A172 cells. To further exclude the possibility of cell-specific inhibition of TNF-α by MgT (50 μM), we also carried out similar experiments in mouse astrocytes (D1A) or microglia (BV2) cells. The results demonstrated that MgT clearly suppressed the expression of TNF-α in D1A and BV2 cells (Figures 3B,C). Similar inhibitory effects of MgT (50 μM) were also observed on the expression of PEN2 and NCT in human SH-SY5Y cells (Figure 3D). To further exclude the possibility of cell-specific inhibition of PEN2 and NCT by MgT (50 μM), we also carried out experiments on mouse n2a cells. As expected, similar results were obtained (Figure 3E). To further examine the roles of Mg^2+^ in the activity of γ-secretases, we examined the protein expression of PS1 and PS2 in SH-SY5Y and n2a cells. However, MgT (50 μM) treatment did not affect the expression of PS1 and PS2 (Supplementary Figure S1). Thus, our experiments reveal the critical role of Mg^2+^ in suppressing the synthesis of TNF-α, PEN2 and NCT in glia and neurons.

### The Pivotal Role of PI3-K/AKT and NF-κB Signaling Pathways in Mediating the Effects of Mg^2+^ on Suppressing the Expression of TNF-α, PEN2 and NCT in Glia and Neurons

**Figure 4:**
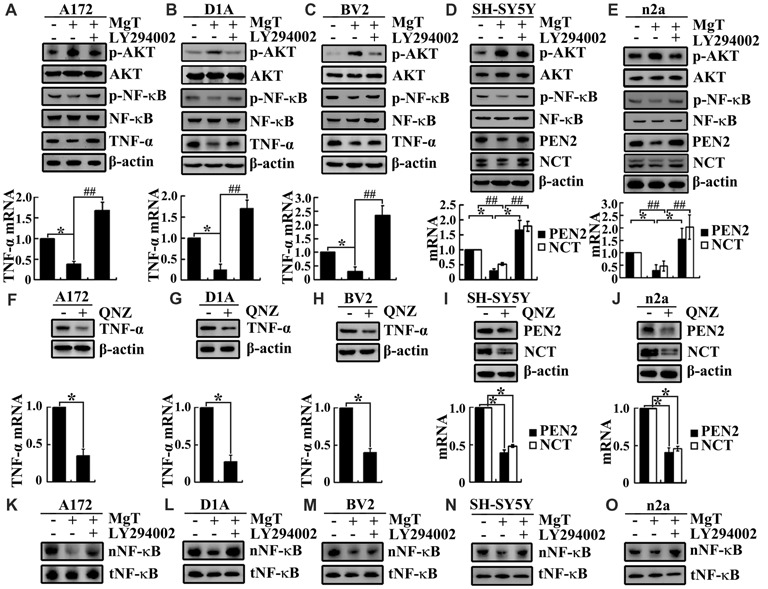
Involvement of PI3K/AKT and NF-κB signaling pathways in regulating the expression of TNF-α, PEN2 and NCT. **(A–C)** A172, D1A and BV2 cells or **(D,E)** SH-SY5Y and n2a cells were treated with MgT (50 μM) in the absence or presence of the PI3-K/AKT inhibitor, LY294002 (10 μM) for 48 h. The mRNA and protein expression of TNF-α, PEN2 and NCT were determined by qRT-PCR and western blots. GAPDH and β-actin served as the internal control. **(A–E)** Phosphorylated AKT, AKT and **(K–O)** nucleus NF-κB were determined by western blots. Equal lane loading was demonstrated by the similar intensities of total β-actin. **(F–H)** A172, D1A and BV2 cells or **(I,J)** SH-SY5Y and n2a cells were treated with the NF-κB inhibitor, quinazoline (QNZ; 1 μM) for 48 h. The mRNA and protein expression of TNF-α, PEN2 and NCT were determined by qRT-PCR and western blots. GAPDH and β-actin served as the internal control. Phosphorylated NF-κB, NF-κB were determined by western blots. Equal lane loading was demonstrated by the similar intensities of total β-actin. The data represent means ± SE of three independent experiments. *p < 0.05, compared with the vehicle-treated controls. ^##^*p* < 0.01 compared with MgT treatment alone.

After observing the suppressive effects of Mg^2+^ on the expression of TNF-α, PEN2 and NCT, we next aimed to reveal the inherent mechanisms. Using glia and neurons, we found that Mg^2+^ treatment induced the phosphorylation of AKT without changing the total protein levels of AKT in glia and neurons (Figures 4A–E). To further elucidate the potential role of PI3-K and AKT pathways in regulating the expression of TNF-α, PEN2 and NCT, we treated glia and neurons with MgT (50 μM) in the absence or presence of an inhibitor specific for PI3-K/AKT, LY294002 (10 μM). The incubation of glia and neurons with LY294002 (10 μM) not only suppressed the MgT-induced phosphorylation of AKT but also reversed the MgT-induced expression of TNF-α, PEN2 and NCT (Figures 4A–E). In line with this discovery, we found that the incubation of glia and neurons with an inhibitor specific for NF-κB, QNZ (1 μM) inhibited expression of the mRNA and protein levels of TNF-α, PEN2 and NCT (Figures 4F–J).

To identify transcriptional regulation of TNF-α, PEN2 and NCT by Mg^2+^, we determined the possible transcriptional factors involved in this process. We found that Mg^2+^ decreased the translocation of NF-κB to the nucleus without changing the total protein of NF-κB in glia and neurons (Figures 4K–O). We next treated glia and neurons with MgT (50 μM) in the absence or presence of the PI3-K/AKT inhibitor, LY294002 (10 μM). The results demonstrated that LY294002 treatment reversed the effect of Mg^2+^ on reducing the accumulation of NF-κB in the nucleus of glia and neurons (Figures 4K–O). Taken together, our data demonstrate the key roles of the PI3-K/AKT and NF-κB signaling pathways in mediating the effects of Mg^2+^ on suppressing the expression of TNF-α, PEN2 and NCT in glia and neurons.

### Mg^2+^ Elevation Inhibits the Expression of TNF-α, PEN2 and NCT in APP/PS1 Tg Mice

**Figure 5:**
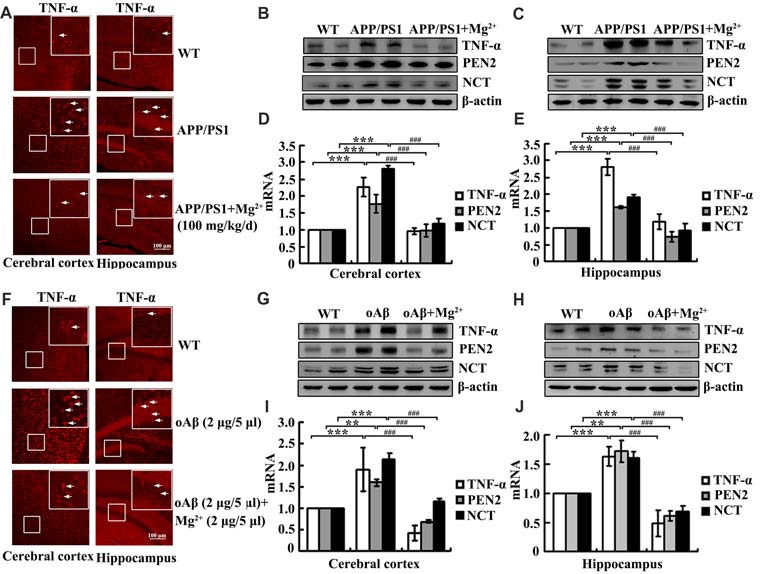
Mg^2+^ elevation suppresses the expression of TNF-α, PEN2 and NCT in APP/PS1 Tg mice. **(A–E)** The APP/PS1 transgenic mice at the age of 4 months old were administered Mg^2+^ (100 mg/kg/d) for 2 months before collecting the brain (*n* = 10). **(F–J)** C57BL/6 mice at the age of 3 months old were injected (i.c.v) with oAβ (2 μg/5 μl) in the absence or presence of Mg^2+^ (2 μg/5 μl). The brains were then collected and sectioned after 24 h. **(A,F)** The immunoreactivity of TNF-α was determined by immunostaining with an anti-TNF-α antibody. These images are representative of ten independent experiments, all with similar results. **(B–E,G–J)** mRNA and protein expression of TNF-α, PEN2 and NCT were determined by qRT-PCR and western blots. Total amounts of GAPDH and β-actin served as an internal control. The data represent the means ± SE of three independent experiments. **p < 0.01 and ***p < 0.001 compared with WT mice or vehicle-treated controls. ^###^*p* < 0.001 compared with APP/PS1 Tg mice or oAβ treatment alone.

Although our data have shown that Mg^2+^ influx suppressed the expression of TNF-α, PEN2 and NCT in glia and neurons, the mechanisms underlying Mg^2+^ elevation *in vivo* have not yet been elucidated. To investigate the relationship between Mg^2+^ elevation and the expression of TNF-α, PEN2 and NCT *in vivo*, immunofluorescence experiments were carried out to determine the regulatory effects of Mg^2+^ on the expression of TNF-α in 6-month-old APP/PS1 Tg mice. Mg^2+^ administration (100 mg/kg/d) for 2 months clearly decreased TNF-α expression in the cerebral cortex and DG region of APP/PS1 Tg mice (Figure 5A). The immunofluorescence results were further validated by qRT-PCR and western blots. Mg^2+^ administration (100 mg/kg/d) for 2 months in 4-month-old APP/PS1 Tg mice inhibited the mRNA and protein expression of TNF-α, PEN2 and NCT (Figures 5B–E). To evaluate the involvement of oAβ in regulating the expression of TNF-α, PEN2 and NCT, intracerebroventricular injection (i.c.v) was employed for treating WT C57BL/6 mice. The immunofluorescence experiments demonstrated that oAβ (2 μg/5 μl) injection (i.c.v) obviously increased the expression of TNF-α in the cerebral cortex and DG region of WT C57BL/6 mice. The upregulation of TNF-α was attenuated by Mg^2+^ (2 μg/5 μl) addition to oAβ-stimulated WT C57BL/6 mice (Figure 5F). These observations were further verified by western blots and qRT-PCR, and similar results were obtained (Figures 5G–J). Thus, it is clear that Mg^2+^ blocks the effects of oAβ on inducing the expression of TNF-α, PEN2 and NCT *in vivo*.

### Mg^2+^ Suppresses the Expression of TNF-α by Decreasing the Activity of Astrocytes and Microglia Cells

**Figure 6:**
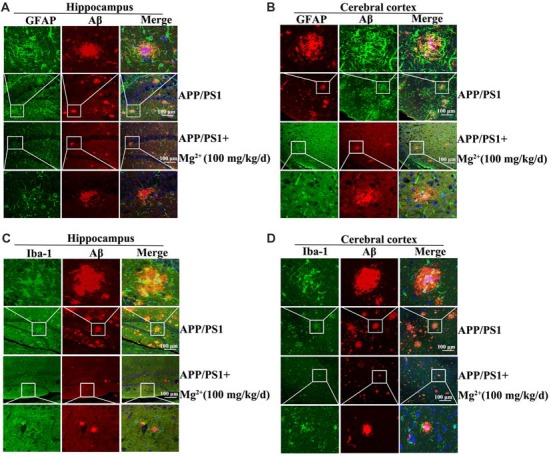
Elevated levels of Mg^2+^ in APP/PS1 Tg mice decrease the activity of glial cells. **(A–D)** The APP/PS1 Tg mice at the age of 4 months old were administered Mg^2+^ (100 mg/kg/d) for 5 months before collecting the brain (*n* = 8). The slices of mouse brains were double-stained with GFAP **(A,B)**, Iba-1 (green) **(C,D)**, or Aβ (red) **(A–D)** antibody before observation using confocal microscopy. These images are representative of eight independent experiments, all with similar results.

In view of the ability of oAβ to stimulate the release of inflammatory cytokines by activating glial cells (Heneka et al., 2015), we next determined the effects Mg^2+^ on the activity of glial cells during the course of AD development and progression. To this end, immunostaining experiments were carried out to measure the activity of astrocytes and microglia cells in APP/PS1 Tg mice. The experiments demonstrated that the activity of astrocytes and microglia cells were markedly elevated in 9-month-old APP/PS1 Tg mice, and this activity was attenuated by Mg^2+^ treatment for 5 months (Figures 6A–D). Of note, these results also validate our prior work (Yu et al., 2015) suggesting that Mg^2+^ treatment decreased the aggregation and deposition of Aβ in 9-month-old APP/PS1 Tg mice. Based on these findings, our data indicated that Mg^2+^ suppressed the expression of TNF-α by decreasing the activity of astrocytes and microglia cells in APP/PS1 Tg mice.

### TNF-α Is Responsible for Upregulating the Expression of PEN2 and NCT in Neurons

**Figure 7:**
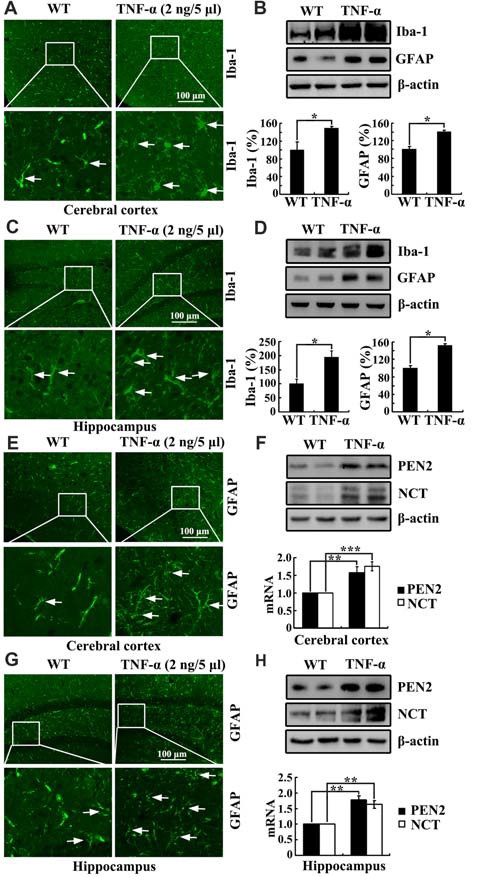
TNF-α stimulates the activity of microglia and astrocytes in APP/PS1 Tg mice. **(A-H)** C57BL/6 mice at the age of 3 months old were injected (i.c.v) with TNF-α (2 ng/5 μl). The brains were then collected and sectioned after 24 h (*n* = 6). **(A,C,E,G)** The activity of astrocytes and microglia were determined by immunostaining with an anti-Iba-1 or -GFAP antibody. These images are representative of six independent experiments, all with similar results. **(B,D)** The protein levels of Iba-1 and GFAP were determined by western blots. The total amount of β-actin served as an internal control. **(F,H)** mRNA and protein levels of PEN2 and NCT were determined by qRT-PCR and western blots, respectively. Total amount of GAPDH and β-actin served as an internal control. The data represent the means ± SE of three independent experiments. *p < 0.05, **p < 0.01 and ***p < 0.001 compared with vehicle-treated controls.

To further study the role of TNF-α in the development of AD, we injected (i.c.v) TNF-α (2 ng/5 μl) into the ventricles of C57BL/6 mice. As expected, TNF-α obviously activated the astrocytes and microglia cells in the cerebral cortex and the DG region of hippocampus compared to that of controls (Figures 7A,C,E,G). In agreement with these results, the protein expression of Iba-1 and GFAP were also upregulated by TNF-α treatment (Figures 7B,D). More interestingly, TNF-α treatment increased the mRNA and protein expression of PEN2 and NCT in the cerebral cortex and the hippocampus (Figures 7F,H). These data illustrate that TNF-α overproduction may accelerate the production and aggregation of Aβ by stimulating the expression of PEN2 and NCT, which are critical for the generation of Aβ.

Taken together, our current investigation reveals that Mg^2+^ ion influx activates the PI3K/AKT signaling pathway and inhibits the NF-κB signaling pathway in glia and neurons, which in turn downregulates the expression of TNF-α, PEN2 and NCT *in vitro* and *in vivo*. The crosstalk between TNF-α and PEN2/NCT could therefore accelerate the production and accumulation of Aβ in APP/PS1 Tg mice (Supplementary Figure S2).

## Discussion

AD is pathologically characterized by the aggregation of extracellular Aβ and intracellular tangles (Shoghi-Jadid et al., 2002). Although Mg^2+^ has been reported to be involved in neuroprotection against AD (Ehmann et al., 1984; Cornett et al., 1998), the underlying mechanisms between Mg^2+^ and AD are still elusive. Thus, this study aimed to decipher the suppressive effects of Mg^2+^ on AD progression. *In* *vitro*, we found that Mg^2+^ inhibited the mRNA and protein expression levels of TNF-α, PEN2 and NCT by activating the PI3-K/AKT signaling pathway and inhibiting the NF-κB signaling pathway in glia and neurons. In agreement with these *in vitro* observations, we found that prolonged administration of Mg^2+^ (100 mg/kg/d) to APP/PS1 Tg mice for 5 months disrupted the crosstalk of TNF-α and PEN2/NCT, which alleviated the development and progression of AD.

Mg^2+^ is strictly regulated and kept at basal levels under physiological conditions. Although the mechanisms of Mg^2+^ downregulation and the role of Mg^2+^ in the brain of AD patients are not fully understood, it has been demonstrated that oral treatment with MgT increases synapse density in aged rats (Slutsky et al., 2010; Li et al., 2014), which is critical for the learning ability of the brain (Jack et al., 2015). In line with these observations, our prior work had found that oral administration of MgT for 5 months improved the cognitive performance of the APP/PS1 Tg mice (Yu et al., 2015). Indeed, Mg^2+^ was reported to concurrently increase α-cleavage and decrease β-cleavage of APP by inhibiting the activity of NMDAR in primary cultured cortical neurons (Hoey et al., 2009). In agreement with this observation, Yu et al. (2010) reported that a high concentration of extracellular Mg^2+^ was able to increase the levels of C-terminal fragment-α (CTFα) and soluble α-secretase cleaved APP (sAPPα) and decrease the concentrations of CTFβ accumulation and Aβ secretion in APP-expressing n2a cells. To further decipher the mechanisms, a number of studies extended these investigations to the activity of β- or γ-secretases. For example, MgT treatment was reported to stabilize β-secretase (BACE-1) expression and to reduce soluble APP and β-C-terminal fragments, which prevented and reversed cognitive deficits and synaptic loss in APP/PS1 transgenic mice (Li et al., 2014). Moreover, our prior work revealed that MgT treatment clearly suppressed the expression of APH-1α/1β, the expression of which enhanced γ-cleavage of APP and resulted in Aβ deposition during the course of AD progression (Yu et al., 2015). In concert with these observations, our data further identified PEN2 and NCT as downstream targets of Mg^2+^ in ameliorating the production and deposition of Aβ in APP/PS1 Tg mice. Along these lines, Mg^2+^ induced its neuroprotective effects on neurons by disrupting the production and deposition of Aβ in a β- or γ-secretase-dependent mechanism.

Apart from the roles of Mg^2+^ in neurons, it was also found to be effective in modulating inflammation. For example, TNF-α was highly induced in alveolar macrophages stimulated by lipopolysaccharide (LPS) in Mg^2+^-deficient medium, but not in Mg^2+^-enriched medium (Shogi et al., 2003; Abbas and Sakr, 2016). In addition, Sugimoto et al. (2012) reported that MgSO_4_ exposure reduced the number of activated monocytes, which produced TNF-α in intrapartum women. Importantly, maternal MgSO_4_ treatment ameliorated the LPS-stimulated inflammation response during pregnancy, which impaired offspring learning ability and memory (Lamhot et al., 2015). In line with these observations, our data extend the prior work suggesting that MgT treatment decreases the expression of TNF-α in glial cells of APP/PS1 Tg mice. Moreover, the PI3-K/AKT and NF-κB pathways have been identified to be critical for mediating the effects of Mg^2+^ on the expression of TNF-α. In agreement with our data, the PI3-K and AKT pathways have also been reported to exert anti-inflammatory functions (Guha and Mackman, 2002; Schabbauer et al., 2004). Importantly, Su et al. (2013) have also reported that MgSO_4_ inhibits the LPS-induced inflammation by activating the PI3-K/AKT pathway and inhibiting the NF-κB pathway.

In view of these observations, we were prompted to investigate the relationship between TNF-α and PEN2/NCT in glia and neurons. The results demonstrated that TNF-α secreted from glia was able to stimulate the expression of PEN2 and NCT in neurons. This finding was substantiated by prior observations showing that TNF-α upregulates APP processing and Aβ deposition in human rhabdomyosarcoma (Keller et al., 2013) and neuroblastoma cells (Blasko et al., 1999). Additionally, our data were further supported by reports demonstrating that anti-TNF-α or inhibition of TNF-α signaling reduced the formation of APs in the brains of APP/PS1 transgenic mouse (McAlpine et al., 2009; Shi et al., 2011). Indeed, Satoh and Kuroda (1999) and Kuo et al. (2008) have suggested that TNF-α regulates APP processing and Aβ deposition by activating PS1 and PS2 in neurons. Although they unfortunately did not extend their analysis to other components of γ-secretase, such as PEN2 and NCT, these *in vitro* and *in vivo* data were clearly in agreement with our data suggesting that TNF-α regulates the formation of Aβ in a γ-secretase-dependent manner in neurons.

In view of the above observations, TNF-α may act as a connector between glia and neurons. Although we found that TNF-α was predominantly expressed in neurons, we could still not negate its existence in glial cells (Figure 2C). Since most of studies regard TNF-α as an exogenous factor in affecting neurons, no related report has shown that TNF-α is highly expressed in neurons to the best of our knowledge. Although we could not find direct evidence showing that the neuronal TNF-α originated from the neuron itself and not glia, Liu H. et al. (2015) reported that Aβ_1–42_ had the ability to stimulate the synthesis of TNF-α in rat hippocampus, which is mainly made up of neurons. In addition, Lv et al. (2014) suggested that the RAGE signalling pathway is important for TNF-α synthesis in Aβ_1–42_-treated BV2 cells. Apart from microglia cells, the injection of Aβ_25–35_ has been used to induce the expression of TNF-α in astrocytes of the rat brain as an experimental model of AD (Díaz et al., 2014; Kim et al., 2014). In addition, these observations also imply that Aβ is the initiator for the AD by stimulating the expression of TNF-α at the early stage of the disease.

More interestingly, we also observed the phenomenon that TNF-α secreted from glial cells translocated and bonded to the neurons (Figure 2C). We therefore clarified the origination of TNF-α in neurons. On the one hand, TNF-α was produced by neurons themselves. On the other hand, TNF-α was also obtained from glial cells. This observation also indicates that TNF-α is not a passive molecule for neurons. Apart from its ability to stimulate the expression of PEN2 and NCT in neurons, TNF-α had an effect on the production and deposition of Aβ. In agreement with our observation, several anti-TNF-α medications have decreased Aβ deposition, cognitive injury and inflammatory response in AD animal models (Russo et al., 2012; Tweedie et al., 2012; Detrait et al., 2014; Gabbita et al., 2015). Importantly, He et al. (2007) reported that disrupting the interaction between TNF-α and tumor necrosis factor type 1 death receptor (TNFR1) inhibited the ability of TNF-α to induce the expression of BACE1, which is critical for Aβ production. Therefore, the efficacy of Mg^2+^ to treat AD is simply due to its ability to disrupt the crosstalk between glia and neurons in a TNF-α- and Aβ-dependent mechanism.

## Author Contributions

XY and P-PG conceived and performed all of the experiments. DZ, Y-YL and TW carried out select experiments. PW along with Z-YW conceived the experiments of this study. PW and Z-YW interpreted the data and wrote the manuscript.

## Conflict of Interest Statement

The authors declare that the research was conducted in the absence of any commercial or financial relationships that could be construed as a potential conflict of interest. The reviewer HB and handling Editor declared their shared affiliation.

## Supplementary Material

FIGURE S1 Involvement of metal ions in regulating the expression of presenilin 1 (PS1)/PS2.

FIGURE S2 Proposed cascade of the signaling events regulating the pathogenesis of Alzheimer’s disease (AD) by magnesium ion (Mg^2+^).
