# Supplementary figures and images for: Magnesium Ions Inhibit the Expression of Tumor Necrosis Factor α and the Activity of γ-Secretase in a β-Amyloid Protein-Dependent Mechanism in APP/PS1 Transgenic Mice
Source: Front Mol Neurosci. 2018 May 30;11:172. doi: 10.3389/fnmol.2018.00172 (PMC5988891; doi:10.3389/fnmol.2018.00172)

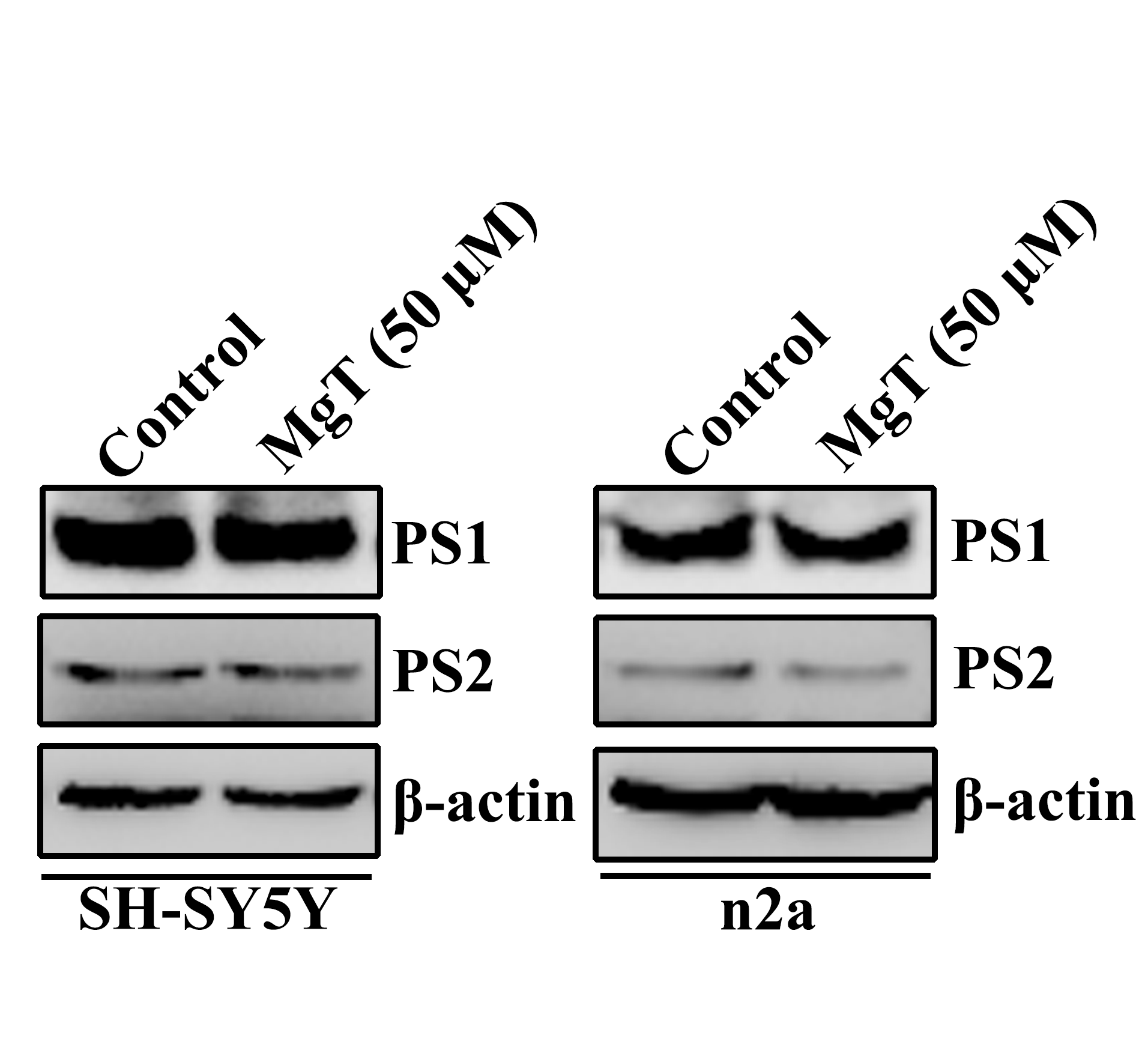

Supplement: FIGURE S1 — Involvement of metal ions in regulating the expression of presenilin 1 (PS1)/PS2. [file Image_1.TIF]

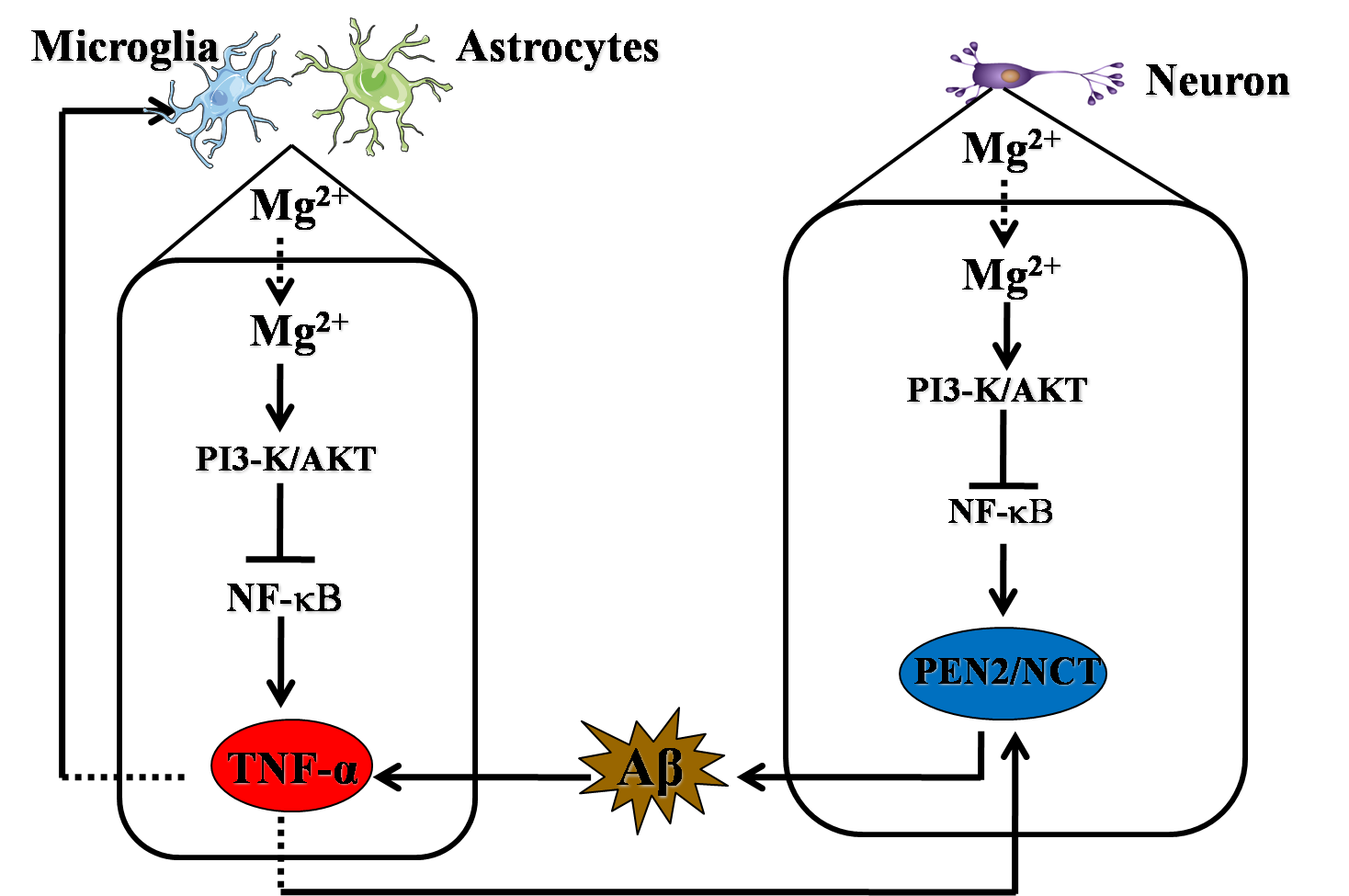

Supplement: FIGURE S2 — Proposed cascade of the signaling events regulating the pathogenesis of Alzheimer’s disease (AD) by magnesium ion (Mg2+). [file Image_2.TIF]
